# Bigh3 silencing increases retinoblastoma tumor growth in the murine SV40-TAg-Rb model

**DOI:** 10.18632/oncotarget.14659

**Published:** 2017-01-14

**Authors:** Nathalie Allaman-Pillet, Anne Oberson, Daniel F Schorderet

**Affiliations:** ^1^ IRO - Institute for Research in Ophthalmology, CH-1950 Sion, Switzerland

**Keywords:** BIGH3, retinoblastoma, cancer, SV40-TAg-Rb mice

## Abstract

BIGH3, a secreted protein of the extracellular matrix interacts with collagen and integrins on the cell surface. BIGH3 can have opposing functions in cancer, acting either as tumor suppressor or promoter by enhancing tumor progression and angiogenesis. In the eye, BIGH3 is expressed in the cornea and the retinal pigment epithelium and could impact on the development of retinoblastoma, the most common paediatric intraocular neoplasm. Retinoblastoma initiation requires the inactivation of both alleles of the RB1 tumor suppressor gene in the developing retina and tumor progression involves additional genomic changes. To determine whether BIGH3 affects retinoblastoma development, we generated a retinoblastoma mouse model with disruption of the *Bigh3* genomic locus. *Bigh3* silencing in these mice resulted in enhanced tumor development in the retina. A decrease in apoptosis is involved in the initial events of tumorigenesis, followed by an increased activity of the pro-survival ERK pathway as well as an upregulation of cyclin-dependent kinases (CDKs). Taken together, these data suggest that BIGH3 acts as a tumor suppressor in the retina.

## INTRODUCTION

BIGH3 protein, also known as TGFΒI (Transforming growth factor-β-induced), is an extracellular matrix (ECM) protein containing four fasciclin-1 (FAS1) domains and a carboxy-terminal Arg-Gly-Asp (RGD) sequence [1–3]. The secreted BIGH3 binds to molecules of the ECM, including fibronectin, laminin and different collagen types [2, 3] and serves as ligand for several integrins, including avβ3 and avβ5 [3–9]. BIGH3 is expressed in most normal human tissues such as heart, liver, pancreas, and skin, but not in brain [1, 7, 10, 11]. BIGH3 expression is strongly induced by TGF-β in several cell lines including human epithelial cells, keratinocytes and fibroblasts [1, 10]. While BIGH3 function in normal tissues is not fully understood, various mutated forms have been associated with corneal dystrophies [12, 13].

BIGH3 also plays a role in cancer progression, with opposite functions of either tumor promoter or suppressor. On one hand, BIGH3 is upregulated in some human cancers like osteosarcoma [14], colon carcinoma and adenoma [15, 16], renal clear cell carcinoma [17, 18] and pancreatic carcinoma [19]. On the other hand, BIGH3 downregulation has been associated with breast carcinoma cells [20], neuroblastoma [21], lung carcinoma [22–24], and mixed lineage leukemia [25]. The variation in BIGH3 expression observed in tumoral cells and reported in the literature has never been associated with *BIGH3* mutations but rather with epigenetic/transcriptional modifications. The metastatic phenotype of lung, prostate and ovarian carcinomas was correlated to *BIGH3* promoter hypermethylation, leading to its silencing [26–29]. Regarding *Bigh3*^−/−^ mice, they were shown to be more susceptible to develop both spontaneous and 7,12-dimethylbenz (α)anthracene-induced skin tumors [30].

The proposed mode of action of the secreted BIGH3 in carcinogenesis is based on its capacity to activate or inhibit integrins through its binding leading to an opposite effect on cell proliferation, adhesion and migration. The reason for this opposite action mode may be dependent on the tissue and/or on the presence of the intact or cleaved form of BIGH3. As a tumor suppressor, BIGH3 inhibits tumorigenesis and reduces the mobility of lung carcinoma cells [20, 31]. At the level of neuroblastoma, BIGH3 represses cell proliferation and invasion [21, 32], while its tumor suppressor effect on osteosarcoma and lung carcinoma cells is mediated by its C-terminal fragment which induces cell apoptosis [33, 34]. When promoting tumorigenesis, BIGH3 is involved in the metastatic process in mice developing colon carcinoma [15], stimulating ovarian carcinoma cell mobility and invasiveness [29]. In hepatocellular carcinoma, BIGH3 induces an integrin-mediated cell invasiveness [35, 36].

We recently reported that BIGH3 is expressed in the retina, where it plays a role in retina maturation [37]. Retinoblastoma (Rb) is a malignant tumor that arises from loss or mutation of both alleles of the RB1 tumor suppressor gene in the developing retina [38]. Loss of both alleles appears, however, to be insufficient to initiate tumor formation, and further genetic and epigenetic modifications may drive to malignancy by finally modulating expression of oncogenes (MYCN, E2F3, DEK, KIF14 and MDM4) and tumor suppressor genes (CDH11, p75NTR) [39–43].

Rb study has been promoted by the development of mouse models that display ocular tumors with Rb characteristics. Unlike children with a defective *RB1* gene, mice with inactivated *Rb1* failed to develop Rb. Rb occurs only when both murine *Rb1* and a related family member gene (*p107* or *p130*) are inactivated. It has been shown that intrinsic compensation affecting the activity or levels of p107 and p130 minimizes the effects of *Rb1* loss, preventing tumorigenesis in mouse [44–47]. Various mouse models have been generated to study Rb. In the SV40 large T antigen transgenic mouse model (SV40-TAg-Rb), the SV40-TAg under the control of the luteinizing hormone β-subunit promoter is expressed in retina, leading to inactivation of RB1, p130 and p107 and promoting Rb formation [48]. In this model, TAg expression in the retina results in aggressive multifocal, bilateral retinal tumors that bear histological and immunological resemblances with human Rb [48, 49]. Rb arises from the inner nuclear layer (INL), and exhibits both Homer Wright and Flexner-Wintersteiner rosettes [48]. In late stage, the tumor fills the vitreous cavity and leads to total detachment and destruction of the remaining retina.

In this study, we determined whether retinal BIGH3 had an impact on Rb development by silencing *Bigh3* in the SV40-TAg-Rb model.

## RESULTS

### Correlation between *BIGH3* mutations and Rb

We reviewed the literature and searched the COSMIC (Catalogue Of Somatic Mutations In Cancer) and ICGC (International Cancer Genome Consortium) databases to identify *BIGH3* point mutations in various cancers and in particular in Rb. Several mutations were observed in various cancers (Table 1), but no functional studies were reported, so far. No mutations in *BIGH3* were reported in Rb.

**Table 1:** Cosmic *BIGH3* gene analysis

Tissue	Point mutations	
	% Mutated	Total number of sample tested
Breast	0.67	2103
Endometrium	2.19	640
Large intestine	2.09	1432
Liver	0.61	1807
Lung	0.54	1835
Pancreas	0.52	1736
Prostate	0.59	1357
Skin	2.19	1189
Stomach	1.62	740

Point mutations in *BIGH3* gene present in human cancer

### Tumor development in SV40-TAg/Bigh3^−/−^ mice compared to SV40-TAg

**Figure 1:**
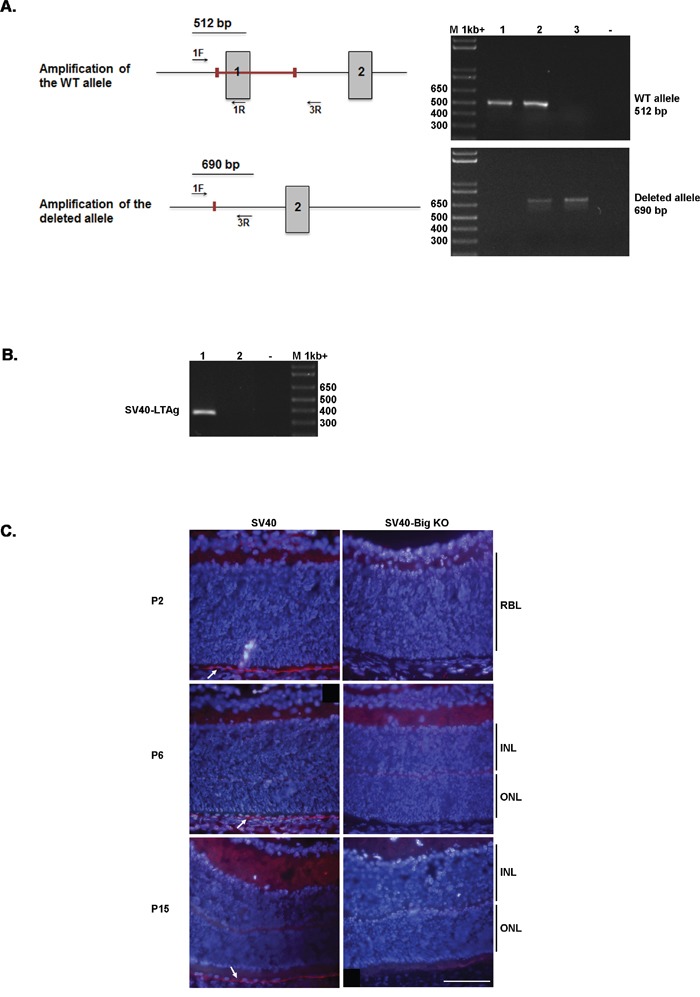
*Bigh3* silencing in RPE of mice retina **A**. PCR approach used to distinguish between *Bigh3* WT, *Bigh3*^+/−^, and *Bigh3*^−/−^ alleles. The figure shows both *Bigh3* WT and deleted alleles with the red bar indicating the deleted region encompassing exon 1. Exon 1 and 2, primers position and amplicons are indicated. PCR product for *Bigh3* WT (1), *Bigh3* ^-/+^ (2), and *Bigh3* ^−/−^ (3) alleles are shown using both primer pairs. M indicated the 1kb ladder, - negative control. **B**. PCR used to detect the SV40-TAg transgene in SV40-TAg (1) and wild type (2) mice. M indicated the 1kb ladder, - negative control. **C**. BIGH3 is detected by immunofluorescence analysis in the retina of SV40-TAg mice at different ages (post-natal day 2, 6 and 15) at the level of RPE, while it is absent from SV40-TAg-Bigh3^−/−^ mice. IH was performed on retina obtained from three different mice at each time point. RBL, retinoblast layer; INL, inner nuclear layer; ONL, outer nuclear layer.

Despite the lack of clear data about *BIGH3* mutations/deletion in Rb, and because of the impact of BIGH3 on the INL maturation at a time when the retinal cells re-enter the cell cycle in our Rb model, we decided to determine whether BIGH3 influences Rb tumorigenesis. To this purpose, SV40-TAg/Bigh3^−/−^ mice were generated by crossing SV40-TAg mice with *Bigh3* deficient mice, two animal models previously described [37, 38]. The genotyping of the SV40-TAg and SV40-TAg/Bigh3^−/−^ mice was performed by PCR using primers specific for the SV40-TAg transgene and for the *Bigh3* gene as described in Materiel and Methods (Figure 1A-1B). We then verified BIGH3 silencing at the protein level by performing immunohistochemistry on the retina of both mice models. As shown in Figure 1C, BIGH3 staining was observed in retina of SV40-TAg mice at the level of the retinal pigment epithelium layer (RPE), while no staining was observed in SV40-TAg/Bigh3^−/−^.

**Figure 2:**
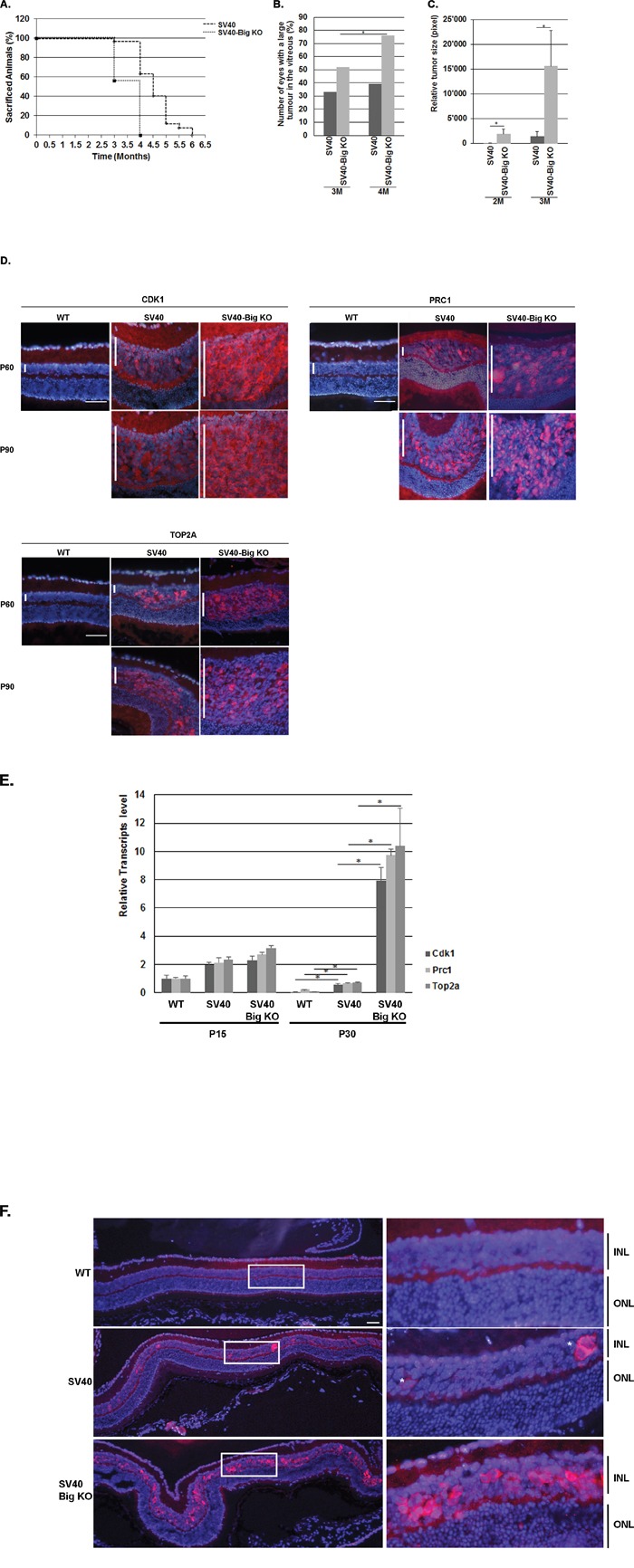
*Bigh3* KO impacted Rb mice viability and tumor incidence and development **A**. Survival curve of SV40-TAg mice developing Rb compared with SV40-TAg/Bigh3^−/−^. **B**. The number of eyes with large Rb visible in the vitreous were counted in SV40-TAg and SV40-TAg/Bigh3^−/−^ mice of 3 and 4 months of age. n= 6 and 56 for SV40-TAg of 3 and 4 months of age respectively and n= 46 and 59 for SV40-TAg/Bigh3^−/−^ of 3 and 4 months of age. *p<0.05. **C**. Estimation of the Rb tumor size in SV40-TAg and SV40-TAg/Bigh3^−/−^ mice of 2 and 3 months of age. n= 5 for each age and phenotype. *p<0.05. **D**. CDK1, PRC1 and TOP2A expression was determined in WT healthy mice (WT) as well as in SV40-TAg and *Bigh3*-null SV40-TAg mice by immunohistochemistry in the retina at P60 and P90 (red staining). Nuclear DNA was revealed with DAPI (blue). n=3 for each age and phenotype. The vertical white bar indicated the INL, inner nuclear layer. The horizontal white line is the scale bar (50 uM). **E**. Expression of *Cdk*, *Prc1* and *Top2a* at mRNA level was determined by quantitative RT-PCR in the retina of WT, SV40-TAg and SV40-TAg/Bigh3^−/−^ mice of 1 month of age. Data are the mean ±SEM of three measurements performed on four different retinas for each age and phenotype. *p < 0.001. **F**. Expression of Ki67 in the retina of 1-month-old WT healthy mice (WT) and in SV40-TAg and SV40-TAg/Bigh3^−/−^ mice. n= 4 for each phenotype. INL, inner nuclear layer; ONL, outer nuclear layer. The horizontal white line is the scale bar (50 uM).

To assess whether BIGH3 plays a role of tumor suppressor or promoter in the retina, a cohort of SV40-TAg (n=85) and SV40-TAg/Bigh3^−/−^ (n=59) was generated and tumor development was studied for up to 6 months. Animals were sacrificed either when reaching end of experimentation period or at an earlier time due to mice illness (large Rb visible in the vitreous, weight loss, bristly fur, apathetic behaviour). In SV40-TAg/Bigh3^−/−^ mice, 100% of the animals were sacrificed between 3 and 4 months of age due to systemic illness, while 63.1% of SV40-TAg mice were still alive at 4 months (Figure 2A). For the animals sacrificed at the age of 3 and 4 months, the number of eyes with large Rb, i.e. tumors visible in the vitreous, were counted. At 4 months of age, 39% of SV40-TAg mice showed large tumors with invasion of the vitreous, against 76% in SV40-TAg/Bigh3^−/−^ mice (Figure 2B).

To assess whether *Bigh3* is involved in early events of Rb tumorigenesis, we studied tumor development at earlier time points. Eyes from 2 and 3 months old euthanized SV40-TAg and SV40-TAg/Bigh3^−/−^ mice were removed and entirely cut to obtain a full set of optic sections. The analysis of every 10th retinal section throughout the entire eye allowed us to estimate Rb tumor size. As shown in Figure 2C, there was a huge increase in tumor size in SV40-TAg/ Bigh3^−/−^ mice with a 56- and 5-fold increase at 2 and 3 months, respectively.

The decrease of SV40-TAg/Bigh3^−/−^ mice viability and the increase of large Rb in *Bigh3* deficient mice highly suggest that *Bigh3* acts as a tumor suppressor in the retina.

To further examine the tumor suppressor impact of *Bigh3* in Rb development, we studied the upregulation of factors known to be upregulated in Rb [50]. At the protein level, CDK1 (cyclin-dependent kinase 1), PRC1 (Protein Regulator of Cytokinesis 1) and TOP2A (topoisomerase II alpha) were rapidly overexpressed at the age of 2 and 3 months in mice retina developing Rb, but never as high as in SV40-TAg/Bigh3^−/−^ mice (Figure 2D). At P30, PCR1 was upregulated 3 fold and CDK1 and TOP2A RNA around 10 fold in SV40-TAg, while all three markers were increased 100 fold in the SV40-TAg/Bigh3^−/−^ retina when compared to wild-type retina of the same age. (Figure 2E).

As tumor size was difficult to estimate at earlier time points, we looked at the expression of Ki67, a marker of proliferating cells at all phases of the cell cycle. In eyes of 1-month-old SV40-TAg mice, only some single cells were positively stained by Ki67 along the whole retina, while in SV40-TAg/Bigh3^−/−^ mice multiple Ki67 positive cells were detected (Figure 2F).

### Apoptosis and ERK activity

**Figure 3:**
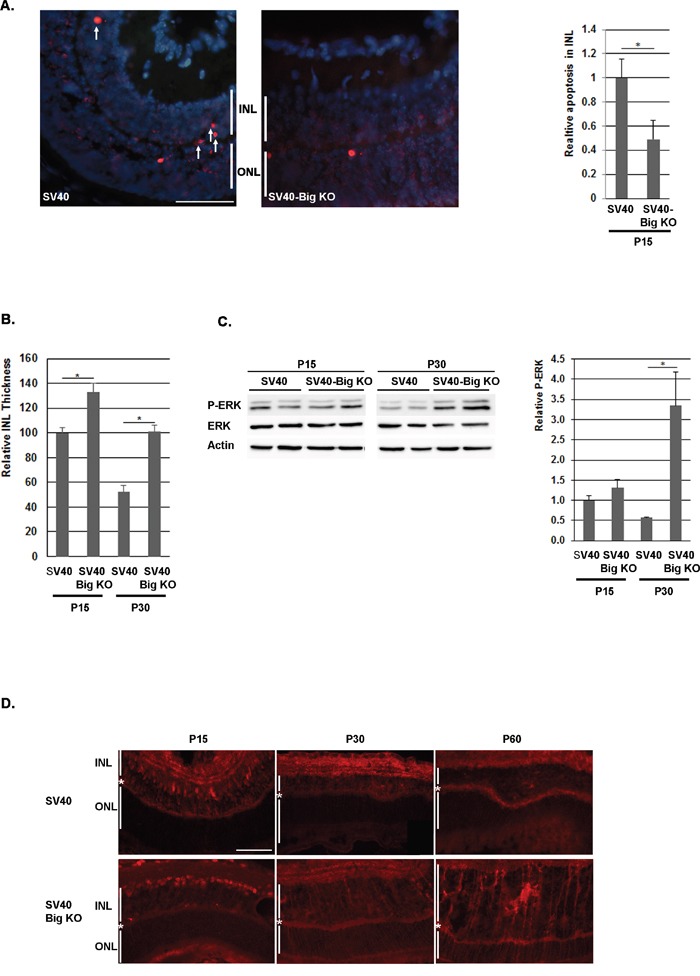
Apoptosis was decreased in the INL of SV40-TAg/Bigh3 KO mice at P15 **A**. TUNEL assay and counting of TUNEL-positive apoptotic cells in the INL showed that apoptotic events were decreased in SV40-TAg/Bigh3 KO retina. Data are the mean +/− SEM of three independent experiments. ONL, outer nuclear layer; INL, inner nuclear layer. The horizontal white line is the scale bar (50 uM). *p<0.014. **B**. INL thickness was measured in SV40-TAg and SV40-TAg/Bigh3 KO retina at day 15 and 30 after birth (P15, P30). Data are the mean ±SEM of three measurements performed on three different retinas. *p < 0.004. **C** and **D**. ERK activity was investigated by Western blotting and immunohistochemistry, respectively in the retina of SV40-TAg and SV40-TAg/Bigh3 KO mice using an anti-P-ERK antibody. In Western blotting, ERK activity was normalized against ERK. Data are the mean ±SEM of three independent experiments. The horizontal white line is the scale bar (50 uM). *p<0.005.

We have previously observed that the absence of BIGH3 in mice retina induced a transient reduction in the apoptotic process in the INL around P10. This anti-apoptotic event seemed to be ERK-dependent and generated a transient increase of the INL thickness around P15, with a higher number of cells compared to WT retina [37]. In the SV40-TAg Rb mouse model, the first cells that express SV40-TAg, which re-enter the cell cycle and from which the tumor originates, are observed between P10 and P15 (Supplementary Figure S1). According to the very early increase in Ki67 positive cells in 1-month-old SV40-TAg/Bigh3 deficient mice compared to SV40-TAg mice, we hypothesized that the increase in the cell number in the INL was sufficient to explain the effect of BIGH3 deficiency on Rb development. To validate this supposition, we first studied apoptosis in the INL at P15. At this moment in time, apoptotic events related to normal retinal maturation are completed in WT retina as well as in retina of *Bigh3^−/−^* mice [37]. As shown in Figure 3A, apoptotic cells were detected in SV40-TAg and SV40-TAg/Bigh3^−/−^ INL with, however, a marked reduction in SV40-TAg/Bigh3^−/−^. We determined whether this reduction in the apoptotic process had an impact on the INL thickness as previously reported in the retina of mice KO for *Bigh3* [37]. *Bigh3* deletion had no impact on the retina organization at P0 and P10 (data not shown). However, we measured a small increase (1.4x) in INL thickness of SV40-TAg/Bigh3^−/−^ retina at P15, which became bigger over time (2 fold at P30) (Figure 3B).

We recently observed that the transient decrease of apoptosis observed in *Bigh3^−/−^* mice at P10 was associated with an increase in ERK activity [37]. At P15, while apoptosis was more pronounced in SV40-TAg retina, ERK activity was identical in both SV40-TAg and SV40-TAg/Bigh3^−/−^ retina (Figure 3C and 3D), suggesting that ERK was not implicated in the apoptosis process at P15. However, the later increase in ERK activity in the SV40-TAg/Bigh3^−/−^ tumor at P30 and P60 (Figure 3D) may suggest that ERK could play a protective role later in tumor development.

**Figure 4:**
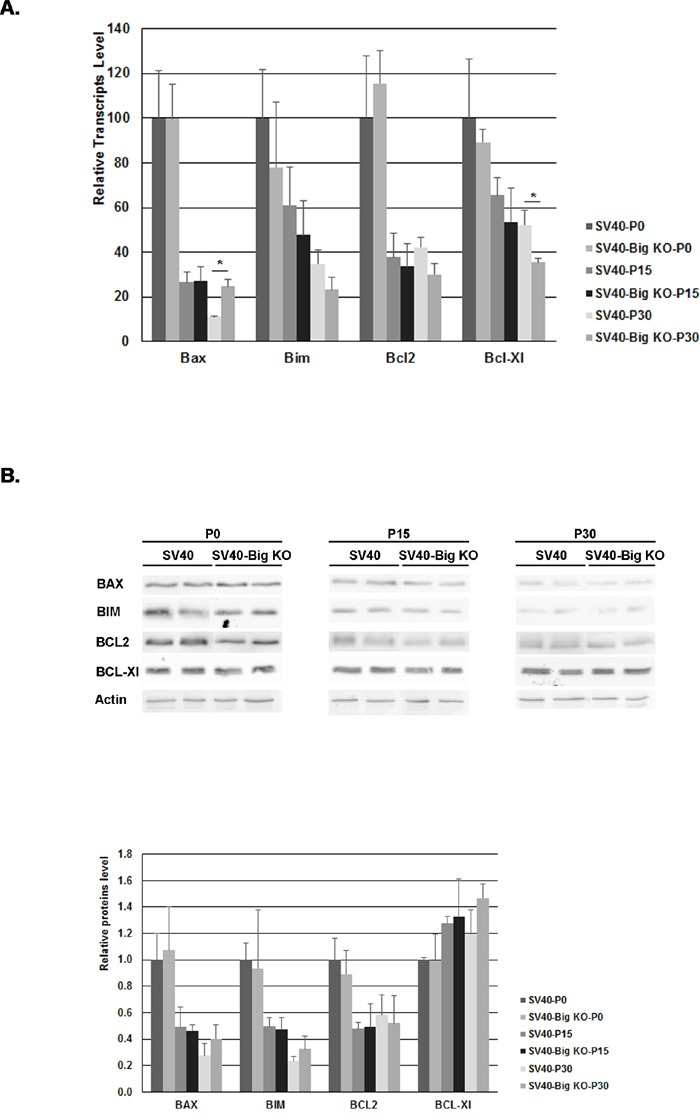
BCL-2 family members content in the retina of SV40-TAg and SV40-TAg/Bigh3 KO mice The content of BCL-2 protein family members was determined at early stage (P0, P15, P30) of Rb development in the retina of SV40-TAg and SV40-TAg/Bigh3 KO mice at mRNA and protein level by quantitative RT-PCR A. and Western blotting B. respectively. Data are the mean ±SEM of three quantitative RT-PCR performed on three different retinas (*p< 0.05), and of Western blot carried out on 4 different retinas for each age and phenotype.

To determine whether the apoptotic modulation was accompanied by a variation in BCL-2 proteins level, the concentration of these major regulators of apoptosis was determined by quantitative RT-PCR (Figure 4A) and by western blotting (Figure 4B). At the transcription level, all *Bcl-2* members were downregulated in the retina at P15, an expression pattern that was previously described in healthy retina [51]. At P30, the pro-apoptotic *Bax* was increased 2 fold while the anti-apoptotic *Bcl-Xl* showed a 1.5 fold decreased in SV40-TAg/ Bigh3^−/−^ compared to SV40-Tag, respectively. At the protein level, the same downregulation in BAX, BIM and BCL2 was observed at P15, while BCL-Xl level kept constant. Surprisingly, the variations observed at P30 between SV40-TAg/ Bigh3^−/−^ and SV40-TAg were not conserved at protein level. These results suggest that the modulation of the *Bcl-2* members at mRNA and protein level did not impact the apoptotic process, suggesting a modulation in the activation of these proteins, which is much more difficult to measure and quantify in a living tissue with a very low percentage of cells triggering apoptosis.

### The regulation of cyclins and cyclin-dependent kinases in SV40-TAg/Bigh3^−/−^ mice

Although the number of cells expressing the SV40-TAg transgene was high in SV40-TAg/ Bigh3^−/−^ retina at P10-P15, following a decrease in the apoptotic process, we could not exclude a modulation in cell proliferation with a potential dysregulation in the cell cycle progression. The recent study of Zhang et al. [30] has indeed reported that CCND1 is overexpressed in *Bigh3^−/−^* cells. In the cell cycle, CCND1 promotes G to S phase progression through binding and activating CDK4/6, which phosphorylate and inactivate RB1 protein, leading to the release of the E2F transcription factors. CCND1 was shown to be upregulated in several cancers, including breast, colon, prostate and hematopoietic malignancies [52, 53].

**Figure 5:**
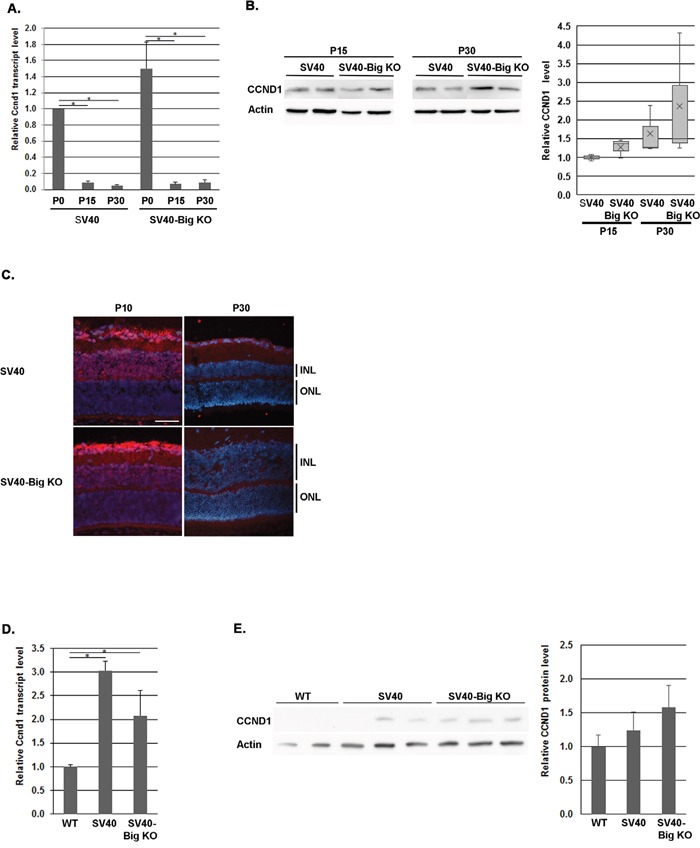
CCND1 expression in *Bigh3*-null SV40-TAg mice Ccnd1 expression was determined at early stage of Rb development at RNA and protein level by quantitative RT-PCR **A**., Western analysis **B**. and immunohistochemistry **C**. in the retina of WT, SV40-TAg and SV40-TAg/Bigh3^−/−^ mice at birth (P0) and at P10, P15 and P30. *p<0.001 in A. Ccnd1 expression was also studied later in large Rb tumor of SV40-TAg and SV40-TAg/Bigh3^−/−^ mice of 4-5 months of age at RNA **D**. and protein **E**. level. n=3 for each age and phenotype. The white line indicated the INL, inner nuclear layer. The horizontal white line is the scale bar (50 μM). *p<0.04 in D.

It is known that CCND1 is the predominant D-cyclin in the developing retina and is highly expressed in RPCs but absent from exited precursors and differentiated cells [54, 55]. By focusing on the first month of life, quantitative RT-PCR analysis showed that *Ccnd1* was highly expressed at birth and was drastically downregulated (around 10 fold) at P15 and P30 (Figure 5A), with few variations between these 2 time points at the mRNA level (Figure 5A), as well as at the protein level (Figure 5B). The immunohistochemistry showed that at P10, as the maturation of the retina was incomplete, CCND1 was still expressed in the INL and GCL, while its level was practically undetectable at P30 in the retina of SV40-TAg as well as of SV40-TAg/ Bigh3^−/−^ mice (Figure 5D). At later stage Ccnd1 was slightly increased at the RNA level (3 and 2 fold at 4 and 5 month, respectively) (Figure 5D), with no significant difference in tumors of SV40 and SV40-Big KO models. At protein level, the tendency was to a non significant increase (Figure 5E). We were therefore unable to detect any upregulation of CCND1 at both RNA and protein level in SV40-TAg/Bigh3^−/−^ model compared to SV40-TAg Rb model in early tumoral stages as well as in large tumors.

**Figure 6:**
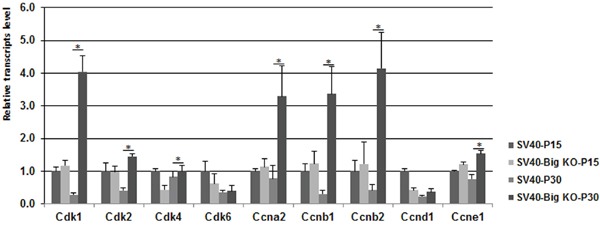
The expression of various Cdks was upregulated in *Bigh3*-null SV40-TAg mice mRNA levels for cell cycle regulatory cyclin-dependent kinases (*Cdk1*, *2*, *4*, and *6*) and cyclins (*Ccna/b*, and *e*) genes were determined by quantitative RT-PCR in P15 and P30 SV40-TAg and SV40-TAg/Bigh3^−/−^ mice retina. Data are the mean ±SEM of three independent experiments performed with the retinal RNA from three different mice. *p<0.006.

To determine whether *Bigh3* loss impacted cell cycle progression by acting on other cyclin-dependent kinases (CDKs) as well as their regulators, the cyclins (CCN), quantitative RT–PCR analysis was performed. As shown in Figure 6, several CDKs and CCNs appeared to be upregulated in SV40-TAg/Bigh3^−/−^ retina compared to SV40-TAg. Cdk1 was increased 14.4 fold, Cdk2 3.5 fold, Cdk4 1.2 fold, Ccna2 4.3 fold, Ccnb1 11.2 fold, Ccnb2 6.5 fold and Ccne1 2.0 fold. These results suggest that the secreted BIGH3 is implicated in maintaining the expression of key regulators of the cell cycle in the retina.

## DISCUSSION

BIGH3 is a secreted ECM protein enhancing cell interactions, and having an impact on various biological processes such as apoptosis, cell growth, cancer, and migration. In the cancer field, BIGH3 has been reported to act as a tumor suppressor as well as a tumor promoter. We recently generated a *Bigh3* deficient mice model that did not display any macroscopic phenotype [37] and was not predisposed to carcinogenesis, unlike a previous different mouse model developing spontaneous tumors [30]. The expression of BIGH3 in the RPE cells led us to investigate whether BIGH3 silencing in the retina could have an impact on Rb, a tumor developing from the INL part of the retina in the mouse.

Rb tumors have a surprisingly low rate of secondary mutations. Whole-genome sequencing of 4 [56] and whole-exome sequencing of 71 tumor samples [57] showed that only BCOR and CREBBP were mutated in 10% and 4% of the cases, respectively. No mutation in *BIGH3* was observed in those samples, which is not surprising as BIGH3 is expressed in RPE cells, a cell type that is not at the origin of Rb. It appears therefore that BIGH3 could impact tumorigenesis at distance.

To confirm this, we demonstrated that BIGH3 acts as a tumor suppressor in the retina during Rb outgrowth by promoting cell death. The evaluation of tumor outgrowth in SV40-TAg and SV40-TAg/Bigh3^−/−^ mice retina allowed us to show an early and constant enhanced tumor progression in *Bigh3* deficient mice leading to a decrease of SV40-TAg/Bigh3^−/−^ mice viability (Figure 2A-2C). During the very early and initial events of tumorigenesis, between P10 and P15, when the first retinal cells re-entered the cell cycle following the expression of the SV40-TAg transgene, apoptosis was induced in the INL of SV40-TAg. This cell death process was, however, decreased in *Bigh3* deficient mice (Figure 3A), leading to an increase of Ki67 positive cells (Figure 2F) re-expressing other markers of dividing cells like CDK1 and PRC1 (Figure 2D-2E). Previous studies have shown that the secreted BIGH3 undergoes carboxy-terminal processing generating soluble RGD peptides able to induce apoptosis [33, 34, 58] by an unclear mechanism. It has been observed that soluble RGD peptides bind integrins at the cell surface, disrupting cell-ECM interactions and leading to anoikis [59]. Another possible way for the RGD peptides to induce apoptosis is to directly enter the cells to activate procaspase-3 [60, 61]. In our mouse model, this non-selective cell death event would seem unlikely as only few cells undergo apoptosis in the INL, suggesting that the RGD peptides modulate apoptosis through cell-specific membrane receptor like the integrins. The integrins belonging to the group of RGD-binding integrins interact with a variety of ECM ligands containing RGD motifs and include α5β1, α8β1, αIIbβ3, and αv-containing integrins [62]. BIGH3 has been shown to interact with α1β1, α3β1, αvβ3, αvβ5, α6β4 and α7β1 integrin heterodimers [63]. While the αvβ3 heterodimer was shown to be absent from mouse retina [64], the expression level of other integrins is not well documented. Looking at the transcript level by quantitative RT-PCR, we observed that the Itgαv transcript was absent from the retina, while Itgβ5 transcript was detected in healthy mice retina, as well as in Rb (Supplementary Figure S2). Additional experimentations are necessary to determine which integrin heterodimers are present at the surface of retinal and cancer cells.

The signaling pathways triggered by interactions between BIGH3 and integrins and leading to the protective role of BIGH3 against tumor development have previously been documented in other cancer models. They showed a role for BIGH3 in later stages of carcinogenesis as anti-metastatic effector, either by enhancing adhesion to ECM proteins [20] and/or by inhibiting the PI3K/AKT signaling pathway [65]. Circulating BIGH3 was also shown to exhibit a tumor suppressor effect by inhibiting tumor vasculature through αvβ3 integrin targeting [66]. We were unable, however, to detect any modification of these different pathways in the retina of our SV40-TAg/Bigh3^−/−^ mice in comparison to SV40-TAg mice (Supplementary Figure S3 and S4).

In *Bigh3^−/−^* retina of SV40-TAg mice of 1 month of age, the pro-survival ERK pathway was activated and the tumor size had already increased (Figure 3C). In our Rb mouse model, the cell of origin of the retinoblastoma was previously shown to display characteristics of mature Müller cells with progenitor properties [67]. Müller cells are specialized glial cells which can undergo reactive gliosis following various retinal injury or disease. In the retina, reactive gliosis is characterized by ERK activation [68]. In our model, development of retinoblastoma seems therefore to trigger gliosis with ERK activation in Müller cells leading to a streaky pattern in immunostaining experiments due to the long shape of these cells, which span the whole width of the retina from the outer limiting membrane (OLM) to the inner limiting membrane (ILM). In the Figure 3D, the streaky pattern was observed only in SV40-Tag/Bigh3 KO retina, but the same pattern can be observed later in SV40-TAg retina, when the tumor size increases.

While the pro-survival factor ERK was activated in SV40-TAg/Bigh3^−/−^ retina, we also observed that BIGH3 knockdown enhanced cell growth by controlling the expression of *Cdk1*, *Cdk2* and *Cdk4* and their regulators cyclin A and cyclin B (Figure 6), which are key players in the regulation of G2 cells progression into M phase.

In summary, our study showed that BIGH3 acts as a tumor suppressor in the developing Rb by first modulating apoptosis, then activating the pro-survival ERK kinase and regulating the cyclins and cyclin-dependent kinases. This tumor suppressor effect is conducted remotely as in the eye BIGH3 is only expressed in RPE cells, while Rb develops in the Müller cells.

## MATERIALS AND METHODS

### Chemicals and antibodies

Species and dilutions of primary antibodies used for Western Blotting and immunohistochemistry experiments were as follows: rabbit anti-CDK1 (1:1′000; Santa Cruz Biotechnology, Santa Cruz, USA), rabbit PRC1 (1:1000, Protein Tech Group, Chicago, USA), rabbit EZH2 (1:1000, Cell Signaling, Danvers), rabbit Ki67 (Abcam, Cambridge, UK), Bax (Santa-Cruz sc-493), Bcl-2 (Cell Signaling, 2876), Bcl-Xl (Cell Signaling, 2764), Bim (Cell Signaling, 2819), rabbit ERK (1:1000, Santa Cruz Biotechnology, Santa Cruz, USA), mouse P-ERK (1:1000, Santa Cruz Biotechnology, Santa Cruz, USA), rabbit CCND1 (1:1000, Santa Cruz Biotechnology, Santa Cruz, USA), mouse anti-AKT (Santa Cruz Biotechnologies Inc., Dallas, TE, USA; sc-5298), rabbit anti-P-AKT (Cell Signaling; #4060), and mouse Actin (1:1000; Sigma, St. Louis, USA).

The secondary antibodies used for western blotting and immunohistochemistry experiments were goat anti-rabbit HRP or goat anti-mouse HRP (Amersham Biosciences, Otelfingen, Switzerland) and Alexa Fluor 594 goat anti-rabbit (Molecular Probes, Invitrogen Inc., Eugene, OR, USA).

### Animal handling

The SV40-TAg (C57BL/6) mice [34] were a gift from Dr. Joan O’Brien. *Bigh3* knock-out mice were obtained as previously described [37]. The animals were maintained and euthanized in accordance with the ARVO Statement for the Use of Animals in Ophthalmic and Vision Research and were approved by the local Committee Office on Use and Care of Animals in Research of the State of Valais, Sion, Switzerland.

SV40-TAg/Bigh3^−/−^ mice were generated as follows: Heterozygous males for SV40-TAg^+/−^ and homozygous mice for *Bigh3^−/−^* were first crossbred to obtain SV40-TAg^+/−^/Bigh3^+/−^ double heterozygous F1 animals. Double heterozygous F1 males SV40-TAg^+/−^/Bigh3^+/−^ and SV40-TAg^-/−^/Bigh3^-/−^ females were mated to generate SV40-TAg^+/−^/Bigh3^-/−^F2 mice used in this study. SV40-TAg and *Bigh3*-specific genotyping was performed by PCR analysis on genomic DNA isolated from ear punch.

### Genotyping by PCR

Ear punch samples were incubated overnight in 400 ul of lysis buffer (10 mM TrisHCl, pH 8.0; 50 mM KCl; 2.5 mM MgCl2; 0.1 mg/ml gelatine; 0.45% NP-40 (v/v); 0.45% Tween-20 (v/v); 6.5 mg/ml proteinase K) at 55° C. After complete lysis, proteinase K was inactivated (10 min at 95° C) and 1.5 ul of this mixture containing the genomic DNA was directly used to perform PCRs.

PCR-based *Bigh3* genotyping through PCR was performed with Gold Taq (Qiagen, Hilden, Germany) using Bigh3-1F, Bigh3-1R and Bigh-3R primers (Table 2) with the following amplification conditions: 5 min of denaturation at 95°C followed by 30 cycles of amplification (95° C 30 sec, 62° C 30 sec and 72° C 1 min) and a final elongation step of 5 min at 72° C. *Bigh3* WT and KO alleles were diagnosed on a 1% agarose gel.

**Table 2:** Primers sequence

Bigh3-1F	5’-GGATTCCTGAATGCCAAGGTG-3’
Bigh3-1R	5’-AGAGCGAGGGTCAGCAGTC-3’
Bigh3-3R	5’-GGGGTACCCGGACCTTAGCTGAGCC-3’
SV40-F	5’-GGAAAGTCCTTGGGGTCTTC-3’
SV40-R	5’-CACTTGTGTGGGTTGATTGC-3’
Ccnd1 forward	5’-GTTGTGCATCTACACTGACA-3’
Ccnd1 reverse	5’-ACAGAGGGCCACAAAGGTC-3’
Cdk1 forward	5’-CCAGGCTCTATCTCATCTTTG-3’
Cdk1 reverse	5’-ATCAGCCAGTTTGATTGTTCC-3’
Cdk2 forward	5’-AGCTCTCCTTGCGTTCCATC-3’
Cdk2 reverse	5’-TCGGATGGCAGTACTGGGTA-3’
Cdk4 forward	5’-TGGATTGCCTCCAGAAGACG-3’
Cdk4 reverse	5’-CTTCAGCGAGGGTTTCTCCA-3’
Cdk6 forward	5’-GCATCGTGATCTGAAACCGC-3’
Cdk6 reverse	5’-CCACGTCTGAACTTCCACGA-3’
Ccna2 forward	5’-CTGTGCACGCTCTGCCG -3’
Ccna2 reverse	5’-CCATGAAGGACCAGCAGTGA -3’
Ccnb1 forward	5’-GCCTGAACCTGAACTTGAAC-3’
Ccnb1 reverse	5’-TCCAGTTGTCGGAGATAAGC-3’
Ccnb2 forward	5’-CTACGTGAAGGACATCTACCA-3’
Ccnb2 reverse	5’-GTCCATGATGGCAATGCACA-3’
Ccnd1 forward	5’-GGTCTGTGAGGAGCAGAAGT-3’
Ccnd1 reverse	5’-GGCCGGATAGAGTTGTCAGT-3’
Ccne1 forward	5’-CACAGCTTCGGGTCTGAGTT-3’
Ccne1 reverse	5’-GCTGACTGCTATCCTCGCTT-3’
Cdk1-F	5’-CCAGGCTCTATCTCATCTTTG-3’
Cdk1-R	5’-ATCAGCCAGTTTGATTGTTCC-3’
Prc1-F	5’-ATCCATCTGTCAGAAGGAGC-3’
Prc1-R	5’-TCCTGAAGTAGCTTCAATTCC-3’
Ezh2-F	5’-CAGCACAAGTCATCCCGTTA-3’
Ezh2-R	5’-CATTCTCTGTCACCATGCAC-3’
Bax-F	5’-CCACCAGCTCTGAACAGA-3’
Bax-R	5’-TGTCCACGTCAGCAATCATCCT-3’
Bim-F	5’-GTCTCAGGAGGAACCTGAAGAT-3’
Bim-R	5’-CAATGCCTTCTCCATACCAGAC-3’
Bcl2-F	5’-GAACTGGGGGAGGATTGTGG-3’
Bcl2-R	5’-GCATGCTGGGGCCATATAGT-3’
Bcl-Xl-F	5’-TGATTCCCATGGCAGCAGTG-3’
Bcl-Xl-R	5’-CAGTTTACTCCATCCCGAAAG-3’
Itgb5-F	5’-CTCCAGGGCCCGTTATGAAA-3’
Itgb5-R	5’-CTACCAGGTCCCTTAGGGCT-3’
Itgav-F	5’-CAGGGACTGAAGTCTTTCGGC-3’
Itgav-R	5’-CTCCCACCAGGAGAAACATCCT-3’

SV40-TAg genotyping through PCR was performed in the same way using the SV40F/SV40R primers (Table 2) with the following amplification conditions: 5 min of denaturation at 95°C followed by 30 cycles of amplification (95° C 30 sec, 55° C 30 sec and 72° C 30 sec) and a final elongation step of 5 min at 72° C.

### Tumor size estimation

At necroscopy, the entire eye was removed and fixed in 4% paraformaldehyde/PBS for 1h followed by cryoprotection in 30% sucrose/PBS overnight. Eyes were then embedded in 30% albumin egg, 3% gelatin (Yazzula buffer) and frozen.

For tumor size study, serial papillary-optic nerve sections (10 um thick) were cut throughout the entire eye, and every 10th section was further stained with DAPI (Invitrogen) to identify nuclei, followed by three washes in PBS, before being mounted in Citifluor AF1 (Citifluor, London, UK). (n=4).

### Immunohistochemistry

Ten μm-embedded frozen retina sections were blocked in PBS with 2% normal goat serum (Sigma) and 0.2% Triton X-100 (Sigma) for 1 h at room temperature (RT) and incubated with primary antibodies in the blocking buffer overnight at 4°C. Sections were then incubated with fluorochrome-conjugated secondary antibody (Alexa-Fluor 594 goat anti-rabbit antibodies) for 1h at RT. Incubation with secondary antibody alone was used as a negative control. Retinas were further counterstained with DAPI (Invitrogen), followed by three washes in PBS, before being mounted in Citifluor AF1 (Citifluor).

The vasculature marker Isolectin-IB4 was added after the blocking/permeabilization step (dilution 1:2000 in the blocking buffer) for 60 min at RT. Isolectin-IB4 was purchased with an Alexa 488 dye conjugate and did not require secondary antibody treatment (Molecular Probes, Eugene, OR).

### Microscopy

Fluorescence microscopy was performed on a Leica DM6000B Microscope, equipped with a Leica DFC365 FX digital camera. Images were captured using the Leica Application Suite (LAS-AF) microscope software. Representative pictures were taken using a 40x/0,85 Leica HC PL-APOCHROMAT objective.

For quantifications of INL thickness and TUNEL assays, pictures were captured in the posterior pole of the retina, once ventrally and once dorsally to the optic nerve head. Three sagittal sections from three and four different animals for each genotype and age were analysed for INL thickness measurement and TUNEL assay respectively.

### Terminal dUTP Nick End-Labeling (TUNEL) of fragmented DNA

DNA strand breaks in cell nuclei were detected by TUNEL assay, according to manufacturer's instructions (Roche) and previously detailed [69]. Briefly, frozen retina sections were permeabilized in 0.1% Triton X-100/0.1% sodium citrate for 2 min on ice and incubated with terminal deoxynucleotidyl transferase (TdT) and fluorescein-12-dUTP or TMR-dUTP for 1 h at 37°C. Retinas were further counterstained with DAPI (Invitrogen), followed by three washes in PBS, before being mounted in Citifluor AF1 (Citifluor). (n = 4)

### Whole cell lysates

Retinas and tumors were dissected under a binocular microscope and isolated cells were put into cold lysis buffer (20 mM Tris-acetate pH 7.0, 0.27 M sucrose, 1 mM EDTA, 1 mM EGTA, 50 mM sodium fluoride, 1%Triton X-100, 10 mM β-glycero-phosphate, 1 mM DTT, 10 mM p-nitrophenyl-phosphate, and antiproteases), and centrifuged at 15,000 rpm for 20 minutes. Supernatants were recovered and stored at –70°C until use. Total protein in cell lysates was quantified using the BCA Protein Assay according to the manufacturer (Life Technologies, Carlsbad, CA, USA).

### Western blotting experiments

Equal quantity of total protein lysates (40 ug per well) were resolved by 8-15% SDS-polyacrylamide gel electrophoresis and electrotransferred onto polyvinylidene difluoride membranes. Nonspecific protein binding was blocked by incubating the membrane with a blocking solution (1x TBS, 0.1% Tween 20, 5% nonfat dried milk powder) for 1 h at room temperature. The blots were then probed with primary antibodies overnight. The immune complex was detected by using a peroxidase-conjugated secondary antibody and the chemioluminescent detection kit according to the manufacturer's specifications (EMD Millipore EMD Millipore, Merck KGaA, Darmstadt, Germany). FUJIFILM Multi Gauge software was used for densitometric analysis.

### RNA isolation

The retinas and tumors were dissected under a binocular microscope, then rapidly isolated in RNAlater (Ambion; Applied Biosystems, Rotkreuz, Switzerland) before being transferred to TRIzol reagent (Invitrogen AG, Basel, Switzerland) and stored at −80 °C until RNA extraction. Total RNA was extracted following the manufacturer's instructions. Both quantity and quality of RNA were determined on a ND-1000 spectrophotometer (NanoDrop technologies, Inc., Wilmington, DE).

### Reverse transcription and quantitative PCR

cDNA synthesis was performed using 2 μg of total RNA in 20 μl reaction volume. This was done using an oligo dT primer according to the manufacturer's manual (Affinity Script; Stratagene; Agilent technologies SA, Morges, Switzerland). For quantitative PCR, cDNA obtained from 50 ng original total RNA was used for PCR amplification using the 2× brilliant SYBR Green QPCR Master Mix (Agilent) with either 250 nM forward and reverse primer pairs, designed to span an intron of the target gene (Table 2). Real-time PCR was performed in triplicate in a Mx3000PTM system (Agilent) with the following cycling conditions: 40 cycles of denaturation at 95°C for 30 sec, annealing at 59°C for 30 sec, and extension at 72°C for 30 sec. Quantitative values were obtained by the cycle number (Ct value) reflecting the point at which fluorescence starts to increase above background at a fixed threshold level. Values obtained for the target genes were normalized with the housekeeping gene Gapdh.

### Statistical analysis

All results were expressed as means ±SEM of the indicated number of experiments. For statistical analysis, the ANOVA test was performed and p values of less than 0.05 were considered to be statistically significant.

## SUPPLEMENTARY MATERIALS FIGURES


