# Supplementary material for: Bigh3 silencing increases retinoblastoma tumor growth in the murine SV40-TAg-Rb model
Source: Oncotarget. 2017 Jan 14;8(9):15490–506. doi: 10.18632/oncotarget.14659 (PMC5362501; doi:10.18632/oncotarget.14659)
Supplement: Supplementary file 1 [file oncotarget-08-15490-s001.pdf]

Bigh3 silencing increases retinoblastoma tumor growth in the murine SV40-TAg-Rb model

SUPPLEMENTARY FIGURES

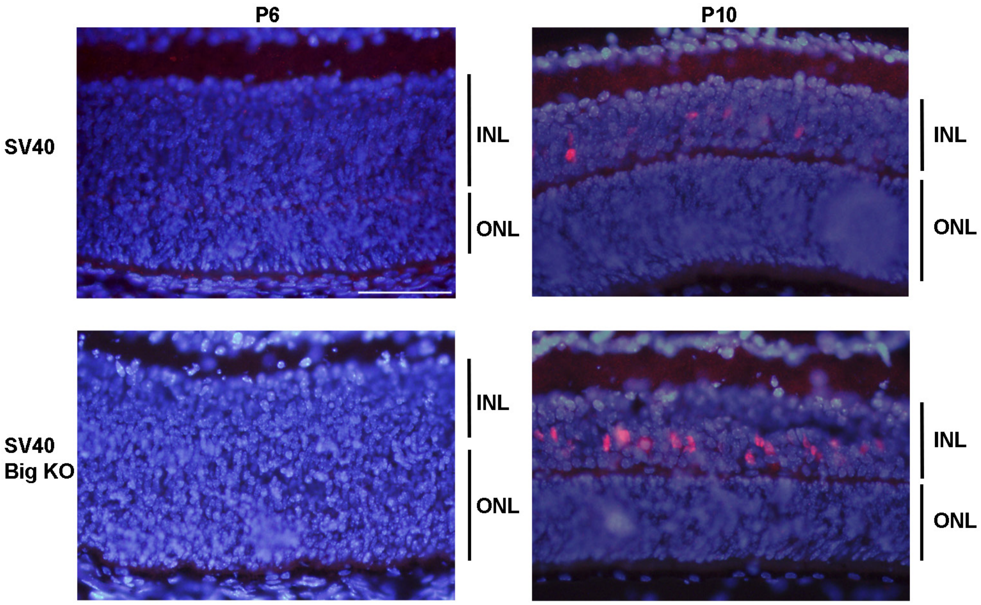

Supplementary Figure 1: SV40-TAg transgene expression in SV40-TAg mice. SV40-TAg expression was investigated by immunohistochemistry in the retina of SV40-TAg mice at P6 and P10 using an anti-SV40-TAg antibody. ONL, outer nuclear layer; INL, inner nuclear layer. The horizontal white line is the scale bar (50 µM).

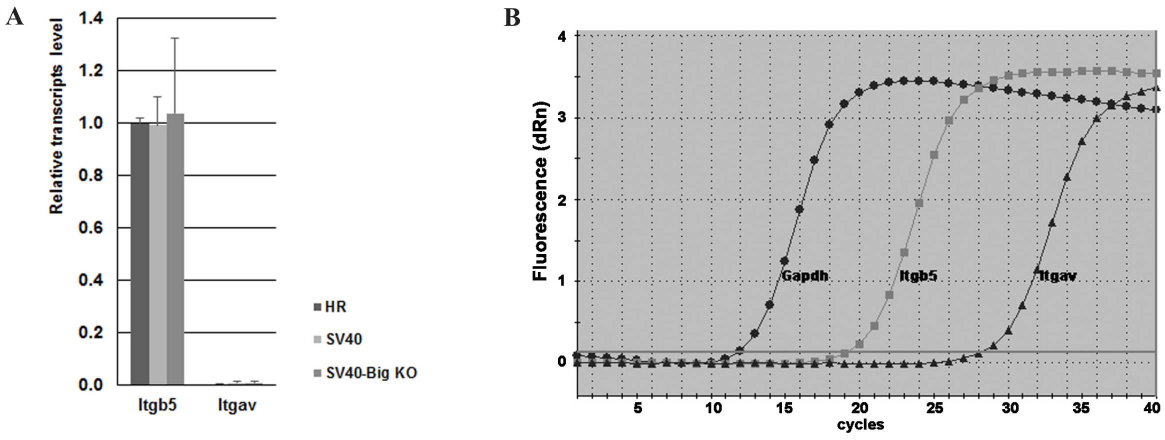

Supplementary Figure 2: Retinal expression of *Itgb5* and *Itgav*. A. Expression of *Itgb5* and *Itgav* was determined at RNA level by quantitative RT-PCR in WT, as well as in large Rb tumor of SV40-TAg and SV40-TAg/Bigh3$^{-/-}$ mice of 4-5 months of age. n=4 for each age and phenotype. **B.** The amplification curve indicated the very low level of Itgva transcript compared to Itgb5 and Gapdh.

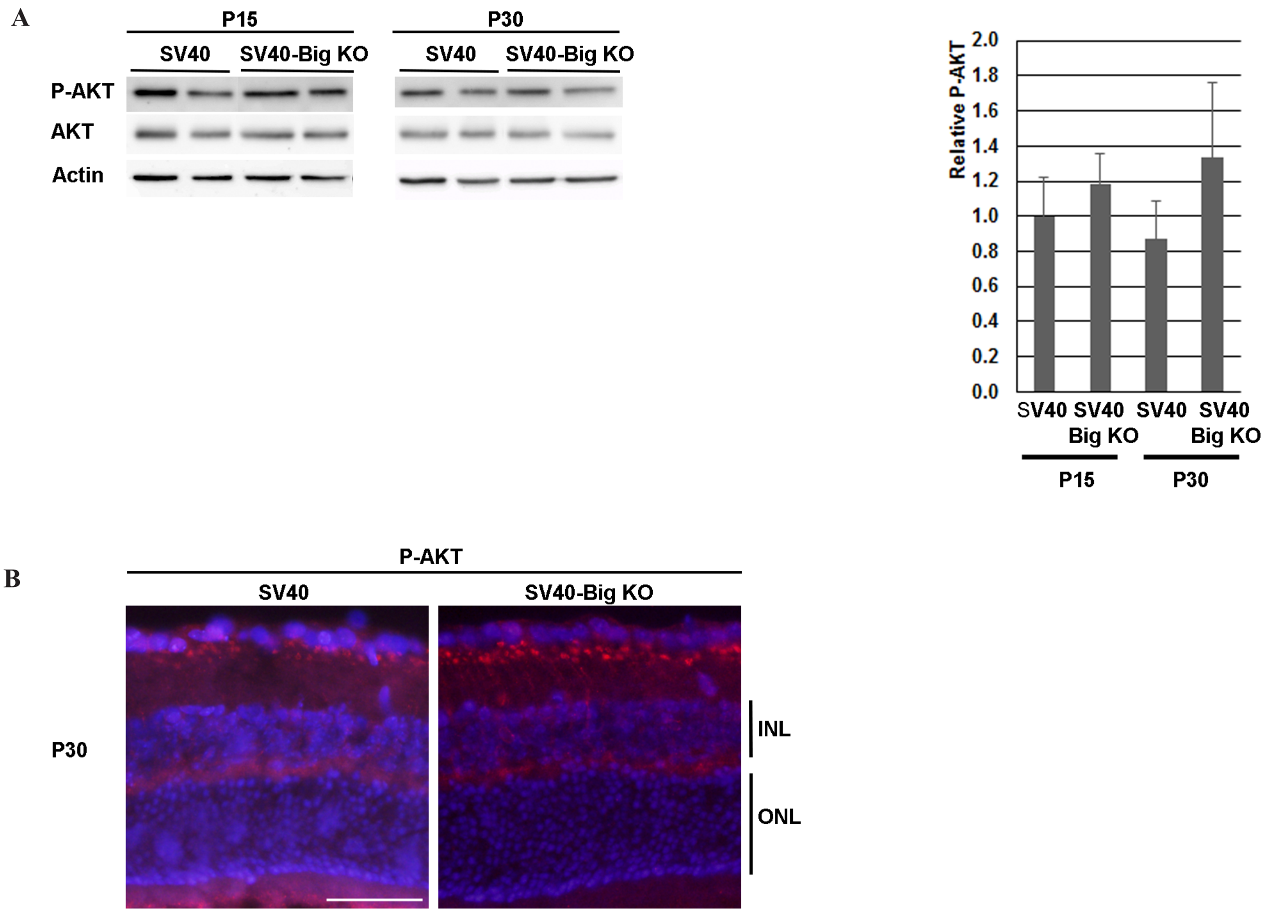

Supplementary Figure 3: AKT activity was investigated by Western blotting A. and immunohistochemistry B. in the retina of SV40-TAg and SV40-TAg/Bigh3 KO mice using an anti-P-AKT antibody. In Western blotting, AKT activity was normalized against AKT. Data are the mean ±SEM of three independent experiments. The horizontal white line is the scale bar (50 µM).

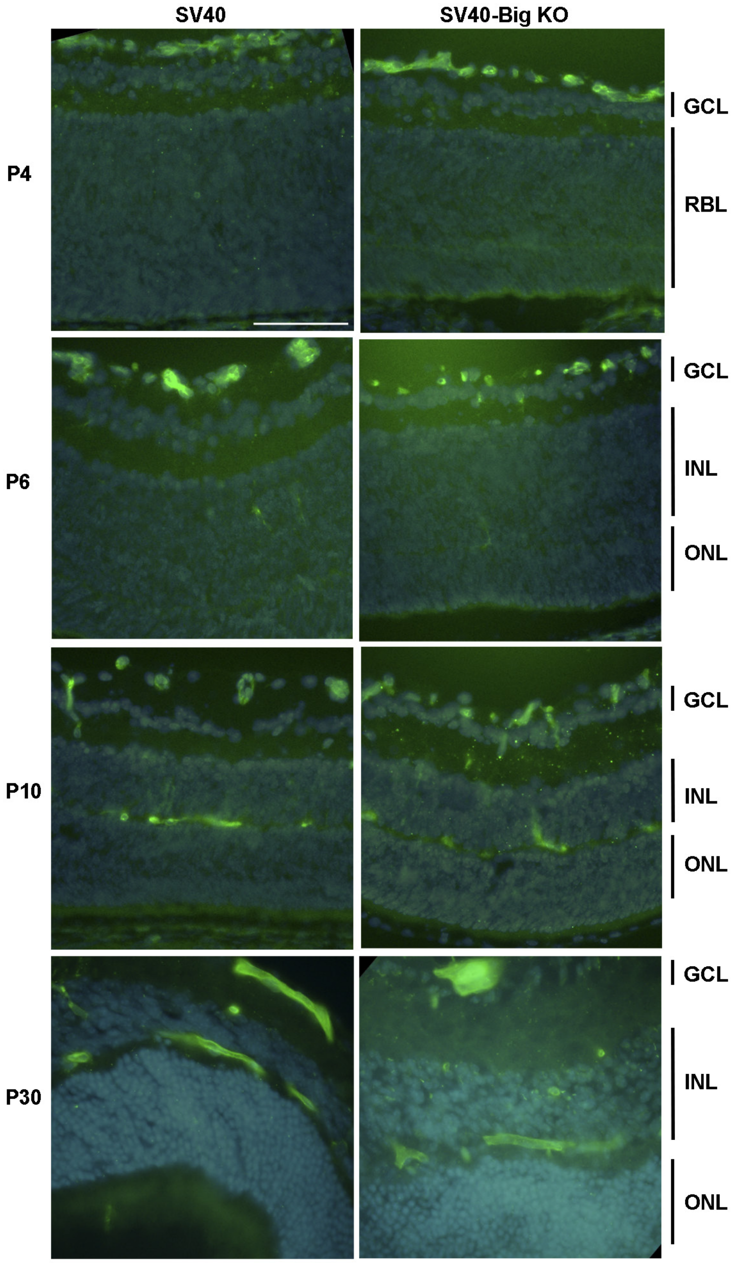

Supplementary Figure 4: Retinal vasculature visualization. To assess postnatal development of retinal angiogenesis, retinal frozen sections of SV40-TAg and SV40-TAg/Big KO mice were stained with FITC conjugate-isolectin B4 (green) which labeled endothelial cells. Nuclei were labeled with DAPI (blue). The blood vessels were first observed in the ganglion cell layer (GCL) at P4 and P6, and their distribution spread later to inner nuclear layer (INL), and inner and outer plexiform layers. No difference was observed between SV40-TAg and SV40-TAg/Big KO retina. n=3 for each age and phenotype. The horizontal white line is the scale bar (50 μM).
